# Application of the theory of planned behavior to model the intention and behavior of tomato growers in pesticide exposure

**DOI:** 10.1016/j.heliyon.2024.e35794

**Published:** 2024-08-03

**Authors:** Amin Pirmoghni, B. Shahmoradi, P. Taymoori, Asghar Bagheri, Parisa Nasrollahi, Zhino Karimi, Farough Mohammadian, Naier Emami, H.J. Choi

**Affiliations:** a Student Research Committee, Kurdistan University of Medical Sciences, Sanandaj, Iran; b Department of Environmental Health Engineering, Faculty of Health, Kurdistan University of Medical Sciences, Sanandaj, Iran; c Department of Health Promotion, Faculty of Health, Kurdistan University of Medical Sciences, Sanandaj, Iran; d Department of Agricultural Management and Water Engineering, Faculty of Agricultural Sciences and Natural Resources, University of Mohaghegh Ardabili, Iran; e Department of Occupational Health Engineering, Faculty of Health, Kurdistan University of Medical Sciences, Sanandaj, Iran; f Department of Agricultural Extension and Education, Faculty of Agriculture, Abu-Ali Sina University, Hamedan, Iran; g Department of Biomedical Science, Catholic Kwandong University, Republic of Korea

**Keywords:** Attitude, Behavioral intention, Sprayers, TPB, Safety

## Abstract

Widespread and indiscriminate use of pesticides has become one of the most important environmental and public health problems around the world. This study was conducted with the aim of applying the theory of planned behavior model to investigate the knowledge, attitude, and behavior of tomato growers in the face of pesticides in agricultural lands located in Kurdistan province, western Iran. We included 300 tomato growers in this study and they filled up a standard TPB questionnaire containing questions about basic information, knowledge, attitude and behavior about pesticides and their actions for disposing of the residual spray solution, washing place of pesticide sprayer, and the disposal of water from washing the equipment. Most of the respondents, 86.7 %, had not participated in promotional classes on how to dispose of the residual solution. Approximately 46.15, 38.46, and 15.39 % of pesticides contained moderately toxic, slightly toxic and practically non-toxic compounds, respectively. More than two-thirds (89 %) of the tomato growers stated that they leave the water from washing the equipment in the field. In addition, among the three variables of TPB, attitudes had the highest score (3.38), which indicated the positive to relatively neutral attitude of farmers towards the safe use of pesticides. These findings can be useful for planners and environmental organizations to make effective interventions to reduce environmental pollution caused by pesticides. Since the incorrect use of pesticides is one of the important environmental and health factors, education and awareness programs can help farmers to consider the correct use of pesticides and environmental protection more.

## 1 Introduction

As one of the main pillars of production in the economic, social and political life of any country, the agricultural sector is of special importance [1]. Considering the growing trend of the world's population and the limitation of arable land, increasing agricultural productivity as the main source of food supply is very important [2]. Tomato (*Solanum lycopersicum* L.) is one of the major food crops around the world and the second most produced vegetable, which is consumed in the diet in the form of soup, sauce, and salad [3]. In fact, it is one of the main sources of flavonoids, vitamin C, K_1_, B_2_, B_9_, potassium, iron, phosphorus and antioxidants such as lycopene and other carotenoids [4]. The world production is about 170 million tons, which is produced about 5.05 million ha [5,6]. The amount of tomato production in Iran is about 6.35 million tons, which is one of the five major fresh export items in terms of weight with 767.9 thousand tons, which is worth 358.45 million US$ [7]. In parallel with the global trend in tomato cultivation, biotic and abiotic stresses reduce the yield of tomatoes in fields [8]. Pest induced stress and diseases put crop productivity at constant risk as it destroys up to 25 % of production [9]. In a study, Gatahi et al. stated that the high cost of inputs, pests and diseases are the major challenges of tomato production at the global level [10]. A low rate of pathogen transmission (e.g. 0.01 %) from seed to seedling can initiate a serious epidemic in commercial tomato fields [11].

The results of recent research works show that there are many pests and diseases (bacterial canker and tomato leafminer moth) that damage both the quality and quantity of tomato production [12,13]. In this regard, a number of factors, cropping barrier, cultural control, physical control, host plant resistance, biological control, biorational pesticides and chemical control have been reported to be effective in controlling pests and weeds [14,15]. However, herbicides, insecticides, fungicides and chemical nematicides are currently used in higher amounts than in the past [16,17]. For example, about 14000 tons of agricultural pesticides, expressed in active ingredients, was applied in Iran during 2012–2014 [18]. These poisons are designed in such a way to disrupt specific biological, metabolic or other aspects of the target organisms [19]. Such aspects are not completely specific to these organisms and therefore similar non-target species may also be affected and lead to ecological imbalance and reduction of biodiversity [20]. The fact is that 99.7 % of the poisons used are wasted after being used in the sprayed area [21].

There are serious concerns about the effects of exposure to pesticides and accidental poisoning or spraying without sufficient protection on the health of farmers [22]. The easy access of the rural population to pesticide products in many low- and middle-income countries has made pesticide poisoning the most common means [23].

Staudacher et al. stated that among the majority of farm owners, there is a minority of migrant workers with low education and no prior training, which has made them a vulnerable group [24]. Therefore, the inappropriate selection of temporary owners and people without commitment to agriculture can lead to an increase in the excessive consumption of chemical fertilizers and pesticides in agriculture [25]. Therefore, people regularly exposed to toxins through inhalation and skin contact in agriculture may reduce the activity of acetylcholinesterase (AChE) in them, which can bring about mental disorders such as depression and suicide attempts [26].

It is necessary to know the controlling factors in order to better deal with the possible risks associated with the use of pesticides [27]. The remaining spray solution after use, sprayer washing places, sprayer washing solution, and expired pesticides are the most important points of creating pesticide residues that need more attention [28]. In many developing countries, farmers may not have access to proper training and equipment for the safe use of agricultural pesticides and waste disposal [29]. In a study, Faryabi et al. investigated that the wrong use of poisons in most cases was related to the weak awareness and attitude of their users [30]. In fact, people's attitude includes a complex set of beliefs, motivations, and experiences of people [31]. Nevertheless, it is necessary to know the level of awareness of farmers and the factors affecting it in relation to pesticides, to avoid its adverse effects on health and reduce poisoning [32]. Abaineh et al. showed that farmers' perception and awareness of the beneficial and harmful effects of pesticides play an important role in their safe consumption [33]. However, in order to change these practices, it seems necessary to know the factors that cause them. Berni et al. concluded that old and experienced farmers do not follow safety instructions [34].

For this purpose, behavioral models can be used to investigate the determinants of behavior related to health and safety among farmers. One of the most effective preventive behavior change models is the Theory of Planned Behavior (TBP) [35]. Diffusion of innovation theory, social cognitive theory, theory of reasoned action, technology acceptance model, and theory of planned behavior (TPB) are of the well-known behavioral theories. The TPB examines the impact of attitudinal components on behavioral intentions and actual behavior that is useful to understand the impact of socio-psychological variable on farmers decision making process. The TPB was presented to explain individuals' behavioral intention to engage in a certain behavior that is a useful approach for explaining human behavior [36,37]. The TPB posits that people intention to perform a specific behavior is determined by three components of subjective norms, attitudes, and perceived behavioral control [36,38]. Attitudes refer to the positive or negative assessments of a behavior [39,40]. According to Ajzen, subjective norms refer to the perceived social pressure to perform or not to perform a behavior [37], and perceive behavioral control refers to perceptions of how much performing a behavior is easy and under his or her control [41,42]. Intention refers to the extent to which an individual tries to perform a behavior, and behavior in turn is under influence of intention and occurs after intention. Understanding farmers' intentions and behaviors concerning pesticide handling and exposure can be resulted in healthy behaviors, promoting the safe pesticide use and protecting the environment. However, little is known about farmers' intention and behavior in this regard. Few studies have examined farmers' intention and behavior in pesticide exposure using the TPB and no study examined this topic among tomato growers in Iran. This study, therefore, aimed to investigate the drivers of tomato growers in pesticide exposure behavior in Kamyaran County of Kurdestan Province, a well-known tomato growing region in Iran. To achieve this objective, the well-known framework of TPB was applied to model tomato growers’ intention and actual behavior. Several theories were presented to explain people decision making behavior.

## 2 Materials and methods

### 2.1 Study area

**Fig. 1:**
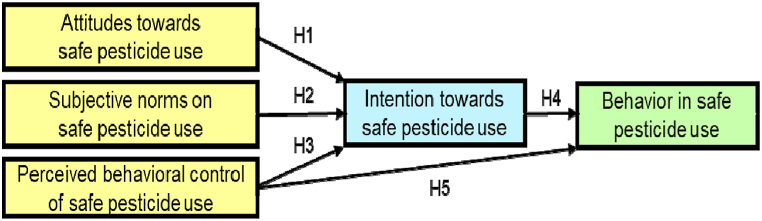
Modified TPB model for safe usage of pesticides (H_1_: There is a relationship between the attitude of tomato growers and the intention to use pesticides safely; H_2_: There is a relationship between tomato growers' mental norms and the intention to use pesticides safely; H_3_: There is a relationship between the perceived behavioral control of tomato growers on the intention to use pesticides safely; H_4_: There is a relationship between tomato growers' intention and actual behavior in the safe use of pesticides; H_5_: There is a relationship between tomato growers' perceived behavioral control and safe use behavior).

This study dealt with the intention and behavior of tomato growers in the safe use of pesticides in Kamiyaran county, Kurdistan province, Iran. The study area of Kamiyaran county with an area of about 1852 km^2^ is located in the south of Kurdistan province with coordinates 34° 47′ 44″ N, 46° 56′ 8″ E and an altitude of 1464 m above sea level. Kurdistan province is one of the most important agricultural regions of Iran due to favorable weather conditions and relatively high annual rainfall. More than 80 % of the residents of this area mainly rely on agricultural activities for their livelihood. Meanwhile, presence of emmigrated farmers from neighbourhood provincesc-in most cases they are interested in increasing crop yeild and having more profits-result in increase of overusage of pesticides and fertilizers as the consequences of their carelessness about the consequences of their practise because they are not the land owners. The average annual temperature and rainfall of the studied area is 5 °C, 572.27 mm [43]. Fig. (1) shows the main hypotheses of this study.

### 2.2 Statistical population

All tomato growers of Kamiyaran County constituted the statistical population of the research. The data of the study was collected using multi-stage random sampling method with proportional assignment. Field observation and interviews with the authorities of the county indicated that about 500 ha of the arable lands are allocated to tomato growing by local people. The statistical population of this study was all tomato growers of Kamiyaran county (N = 700). The studied sample was calculated according to previous studies and considering the maximum error of P = 0.5 and d = 0.06 (accuracy), and with 95 % confidence. We selected 30 villages were from four districts in the region, and farmers were selected from each village in proportion to their population. Those villages that cultivated tomatoes were selected, and the other villages that did not grow this product were excluded from the sample. From each village, 10 farmers were randomly selected among the tomato's growers. Hence, the sample size was 277 people, and for more accuracy, 300 samples was considered [44,45]. The entry and exit criteria for the study included: the entry criteria was voluntary; those tomato growers who were farmers and had land and were also interested in this study were selected. Exclusion criteria: During the study, if something happened to the farmer (he became ill), he did not want to cooperate, or we found out that the farmer was not the party or did not have information in this field, we removed them from the sample and randomly selected another person.

### 2.3 Sampling tool

The tomato growers of each village were interviewed according to their families. In order to collect data, the objectives of the research were first told to the farmer, and then face-to-face interviews were used. Standard TPB questionnaire was used as a data collection tool for research purposes. The questionnaire includes various parts such as demographic characteristics of farmers, methods of pesticide waste disposal as follows. Disposing of the remaining spray solution, the washing area of sprays and equipment, and disposing of the water used to wash the equipment. Use of personal protective equipment (PPE) and barriers affecting the use of PPE in transporting pesticides (8 items), behavior (8 items), attitudes (11 items), perceived behavioral control)PBC((5 items), mental norms (5 items) and intention (5 items). Most of the items in the questionnaire were evaluated on a five-point Likert scale (from never = 1 to very much = 5). A modified version of the TPB standard questionnaire which was validated and used by Bagheri et al. [46]. was used for data collection with necessary changes based on the objectives of the study. Prior to field study a panel of field agricultural experts of Kurdistan province, and university staff from faculty of agriculture and faculty of health of the university of Mohaghegh Ardabili, Ardabil, and Kurdistan University of Medical Sciences, respectively confirmed the content validity on the research tool.

To determine the reliability of the questionnaire, Cronbach's alpha was used; values > 0.70 indicate acceptable reliability. For the pre-test of the research tool and measurement of the alpha value, a preliminary study was conducted using a sample of 30 tomato growers outside the region. Alpha coefficients of 0.702, 0.818, 0.767, 0.774, and 0.931 were obtained in the preliminary study for behavior, attitude, PBC, mental norms, and intention, respectively, which shows the high reliability of the research tool [47].

### 2.4 Data analysis

This study was conducted in 2023. The preliminary study was conducted in February 2022, while the data collection was completed in 4 months (September to December 2023) and in this period, 300 samples were completely completed. The collected data were coded and analyzed using SPSS (Ver. 24) and Lisrel (Ver. 8.8) software. To measure the effect of independent variables on the dependent variable, mean comparison tests, correlation and structural equation modeling (SEM) were used. SEM and path analysis were used to test hypotheses using Lisrel software. Due to the non-normal distribution of quantitative data, Spearman's correlation coefficient was used.

## 3 Results

### 3.1 Name and toxicity class of pesticides used by tomato growers

Table (1) shows the name and toxicity class of pesticides used by tomato growers in this study. The most common pesticides were Fungicide (30.77 %), Insecticide (30.77 %), Herbicide (23.08 %) and Acaricide (15.38 %). Compared with WHO toxicity classes, approximately 15.46, 38.46, and 15.39 % of pesticides contained moderately toxic, slightly toxic, and practically non-toxic compounds, respectively. Table (2) is the types of fertilizers used by farmers to grow tomatoes, most of which are used to strengthen this product. The information provided in Table 1, Table 2 were obtained through field interviews with tomato growers in the study area.

**Table 1:** Name and toxicity class of pesticides used by tomato growers in this study.

Trade Name	Common Name	Chemical Formula	Groupe	Toxicity	WHO class
Dagonis	Difenoconazole (50 g/l)+ Xemium (Fluxapyroxad),(75 g/l)	C_19_H_17_Cl_2_N_3_O_3_	Fungicide	LD50 rat (oral) > 2000 mg/kg	III
Topas	Penconazole	C_13_H_15_Cl_2_N_3_	Fungicide	LD50 rat (oral) > 2125 mg/kg	III
Stroby	Kresoxim-methyl	C_18_H_19_NO_4_	Fungicide	LD50 rat (oral) > 5000 mg/kg	U
Signum 33.4%WG	pyraclostrobin and boscalid	C_18_H_12_CI_2_N_2_O	Fungicide	LD50 rat (oral) > 500 < 2000 mg/kg	III
Dursban	Chlorpyrifos	C_9_H_11_CL_3_NO_3_PS	Insecticide	LD50 rat (oral) 135 mg/kg	II
Rogor and B-58	Dimethoate	C_5_H_12_NO_3_PS_2_	Insecticide	LD50 rat (oral) > 387 mg/kg	II
Ripcord	Cypermethrin EC40 %	C_22_H_19_Cl_2_NO_3_	Insecticide	LD50 rat (oral): 250–4150 mg/kg	II
Decis	Deltamethrin EC2.5 %	C_22_H_19_Br_2_NO_3_	Insecticide	LD50 rat (oral):135–5000 mg/kg	II
Gramaxon	Paraquat	C_12_H_14_Cl_2_N_2_	Herbicide	LD50 rat (oral): 129 mg/kg	II
MCPA	2-methyl-4-chlorophenoxyacetic acid	C_9_H_9_ClO_3_	Herbicide	LD50 rat (oral) 700 mg/kg to 1160	II
Cruz	Nicosulfuron	C_15_H_18_N_6_O_6_S	Herbicide	LD50 rat (oral) > 5000 mg/kg	U
Nissorun	Hexythiazox	C_17_H_21_ClN_2_O_2_S	Acaricide	LD50 rat (oral) > 5000 mg/kg	III
Neoron	Bromopropylate	C_17_H_16_Br_2_O_3_	Acaricide	LD50 rat (oral): 5000 mg/kg	III

**Table 2:** The types of fertilizers used by tomato growers as conditioner or strengthener.

Fertilizer name	Application
Humi King	Root strengthening
Amino acid powder fertilizer	Strengthening flowers and bushes
Biofertilizer (Tisan Root Start)	Growth enhancer and anti-stress
Organamin Amino Acid	Anti-stress, strengthening and increasing the productivity of plants
Flower Set	Strengthening flowering - preventing flower drop
BioFert brand TerraLink's	Crop coloring improver
Fe 6 %	Better growth and increased fruiting, plant chlorophyll production
fish fertilizer	Root strengthening, more plant performance
Flourish Cu14 %	Preventing plant wilting, photosynthesis and plant respiration and increasing plant tolerance against fungal disease
Nutritech Humic Acid 123	Reducing the stress caused by transplanting - improving rooting
Foliar soap	Along with pesticides and foliar fertilizers to wash the plant and reduce the population of pests

### 3.2 Social and economic characteristics of the respondents

The average age of the studied samples was 42.28 yr with a standard deviation of 8.74 and the average size of the household was 4.12 with a standard deviation of 1.26. The highest and lowest observed ages were 58 and 22 yr, respectively. Among the studied samples, 41 people (13.6 %) were illiterate, 51 people (17 %) had primary education, 111 people (37 %) had middle school education, 39 people (13 %) had high school education, and 58 (19.4 %) had university education. Most of the tomato growers had 6–15 yr of tomato cultivation experience. Most of the tomato growers, 86.7 %, had not participated in extension classes on how to dispose of the residual spraying solution (Table 3).

**Table 3:** Characteristics of tomato growers.

Characteristics	Number
Gender	
Male	300 (100 %)
Age (yr)	
≤26	[8] (2.7 %)
27-34	[48] (18.3 %)
35-42	(81) (27.0 %)
43-50	(101) (33.7 %)
≥51	[48] (18.3 %)
Education	
illiterate	[41] (13.6 %)
Elementary school	[49] (17 %)
Middle School	(111) (37 %)
High school	[39] (13 %)
graduated of higher education	[50] (19.4 %)
tomato cultivation experience (yr)	
≤5	[51] (17.6 %)
6-15	(130) (43.3 %)
16-25	[47] (15.7 %)
≥26	[52] (23.4 %)
spraying experience (yr)	
≤5	[36] (12 %)
6-15	(144) (48 %)
16-25	(95) (31.7 %)
≥26	[25] (8.3 %)
number - family	
≤3	(93) (31.0 %)
4-5	(176) (58.7 %)
6-7	[26] (8.7 %)
≥8	[5] (1.7 %)
Information on pesticides	
No	[7] (2.3 %)
Low	[28] (9.3 %)
Average	(178) (59.3 %)
very	(87) (29.1 %)
Training about pesticide	
Yes	[40] (13.3 %)
NO	(260) (86.7 %)
What is your source of learning on pesticides	
experimentally	(108) (36 %)
Pesticide retailers	[52] (23.2 %)
Other farmers	(87) (29.1 %)
TV/Internet/papers/Books	[35] (11.7 %)

### 3.3 Pesticide residual disposal

According to Table 4, 27.3 % of the pesticide residues are sprayed on the treated area, 4.6 % are stored in the field, 51.1 % are used in another tomato field, and only 17 % of the respondents store it in a special warehouse. Meanwhile, 15 % of the respondents wash the sprayer by the river, and the least of them (13.3 %) in the yard, 32.7 % near the water source and 39 % in the field water source. 6.3 % leave the water from washing into the running water, 4.7 % release it inside the water channel, and most of them (89 %) leave it on the sprayed ground.

**Table 4:** Management of pesticide residual.

Variable	Category	percentage
Disposal of residual solution from spraying	Re-spraying the treated land	27.3
	Use in another tomato farm	51.1
	Storage in farm	4.6
	Storage in a special warehouse	17
Washing place for sprayer and spraying tools	Riverside	15
	Home yard	13.3
	Near the water source	32.7
	Sprayed farm	39
Disposal of water used for washing sprayers	into running water	6.3
	Inside the water channel	4.7
	Sprayed land	89

### 3.4 Usage of PPE

The results tabulated in Table 5 shows that special spraying clothing has the highest importance with 87 %. After that, personal protective glasses are in the next category with 73.7 %. In general, Table 5 shows the percentage of each criterion of using PPE.

**Table 5:** Percentage of use of PPE and reasons for not using it.

Sl. No.	Do you use the following PPE?	Yes	No	What is your reason for not using this PPE?					
				A: Uncomfortableness	B: Expensiveness	C: Time-consuming	D: Inaccessibleness	E: It is not necessary	F: all cases
1	Mask	76.7	23.3	15	–	–	–	–	8.3
2	Glasses	26.3	73.7	20	–	–	21.3	–	32.4
3	Hat	52.7	47.3	–	–	–	–	21	26.3
4	Boots	47	53	40	–	–	–	–	13
5	Gloves	67.3	32.7		–	–	–	–	32.7
6	Long pants	90.7	9.3		–	–	–	9.3	–
7	Long shirts	37.7	62.3		–	–	–	45.3	17
8	Spraying clothes	13	87		–	–	–	33.7	53.3

### 3.5 Structures and statements of TPB

According to Table 6, among these 11 criterion items, "At the time of spraying, the pesticide solution should be prepared according to the area under the crop" achieved the highest importance with an average of 4.62, (overall average was 3.38). Meanwhile, among the 5 criteria used for the subjective norms of the tomato growers towards the empty containers of pesticides, the criterion of "The agricultural experts prohibit us from excessive use of pesticides" with an average of 22.3 obtained the highest importance among other components (overall average of 2.34). As observed, among the five criteria for the perceived behavioral control of tomato growers regarding the pesticide residue solution, the criterion "I keep the residual pesticide out of the reach of children" with an average of 4.68 obtained the highest importance among other components (overall average is 2.7). Among five intention criteria, the criterion "I intend to buy poisons as needed" with an average of 4.20 obtained the highest importance among other components (overall average 2.57). Finally, among the eight criteria used for the behavior of tomato growers towards the pesticide residue solution, "I keep the pesticide residue in a suitable place" with an average of 4.87 had the highest importance (overall average 2.6).

**Table 6:** TPB construct and statement of tomato growers.

	TPB construct and statement	Mean	SD
Sl. No.	Attitude	3.38	0.53
1	We can flush pesticide residue down the sink/toilet*^.^	1.05	0.30
2	We must use the remaining solution of pesticide in the field to empty the spray tank, even if this amount of pesticide is more than the product needs*^.^	3.68	1.29
3	We can mix old pesticide residue with new pesticide and reuse it*^.^	1.71	1.06
4	Pesticides available in the market have little effect and to ensure the result, they should be consumed more than the recommended amount*^.^	2.78	1.20
5	It is dangerous to use more pesticide solution than recommended in spraying fields*	3.43	1.32
6	We should buy pesticides as needed	4.06	1.04
7	We should use pesticides as much as needed	4.49	0.79
8	At the time of spraying, the pesticide solution should be prepared as much as the area under the crop.	4.62	0.67
9	Pouring unused pesticide solution in the field is harmful to the environment.	3.93	1.36
10	Pouring unused pesticide solution in the field is harmful for human and animal health.	3.85	1.38
11	Pesticide solution left in the environment can leak into the surrounding soil and pollute surface and groundwater.	3.57	1.59
Sl. No.	The subjective norms	2.34	0.75
1	Agricultural experts forbid us to use too much pesticides on crops.	3.22	1.41
2	My friends and acquaintances leave pesticide residues in the farm environment.	1.84	1.15
3	Pesticide sellers prohibit mixing residual pesticides with other poisons.	3.13	1.33
4	Pesticide sellers recommend that we return the remaining pesticides to them.	1.04	0.40
5	My family forbids me from buying more pesticide than the farm needs.	2.47	1.40
Sl. No.	Perceived behavioral control (PBC)	2.7	0.48
1	I return the remaining pesticides to the pesticide sellers.	1.02	0.18
2	I buy pesticides as needed.	4.19	0.99
3	I keep the remaining pesticides in a closed container and in a suitable place.	1.99	1.42
4	I keep the remaining pesticides out of the reach of children.	4.68	0.84
5	I dump the pesticide residue in the field.	1.64	1.06
Sl. No.	The intention	2.57	0.66
1	I plan to give the residue of my pesticide to the neighboring farmer for consumption in the field.	2.43	1.21
2	I plan to buy pesticide as needed.	4.20	0.95
3	I want to store the pesticide residue in a separate storage.	2.27	1.32
4	I plan to return the pesticide residue to the pesticide sellers.	1.29	0.71
5	I have planned for proper disposal of my pesticide residue.	2.68	1.46
Sl. No.	The behavior	2.6	0.33
1	I keep the pesticide residue in a suitable place out of the reach of children	4.87	0.45
2	I buy pesticides as recommended by experts	4.00	1.16
3	I spray pesticides according to the recommendations of the label or experts	4.09	0.94
4	I continue spraying until the sprayer solution runs out.	3.73	1.22
5	I pour the remaining pesticide in the river/water canal and wash the sprayer	1.23	0.63
6	I put the surplus pesticide in a separate container and keep it for the next spraying	1.73	1.20
7	I pour the remaining sprayer solution in the field and wash the sprayer.	1.79	1.19
8	I give my surplus pesticide solution to other farmers	2.35	1.29

### 3.6 Model fitness indices

In the present research, in order to identify the influencing factors on the behavior of tomato growers, structural equation modeling was used, and fit indices were used to evaluate the fitness of the measurement model. Table (7) shows these indicators along with the acceptable level and the observed value. Based on the specified level, all the suitability indicators of the research model are in good condition.

**Table 7:** Model fitness indices.

Index	Appropriate	Reported
χ^2^/df	1≤ & ≤3	1.70
SRMR	0.10≥	0.090
RMSEA	0.05≥	0.048
GFI	0. 90≤	0.94
AGFI	0.90≥	0.92
NFI	0.90≥	0.96
NNFI	0.90≥	0.98
CFI	0.90≥	0.94
IFI	0.90≥	0.96

SRMR: standardized root mean residual, RMSEA: root mean square error of approximation, NNFI: non-normed fit index, NFI: normed fit index, CFI: comparative fit index, IFI: incremental fit index, GFI: goodness of fit index, AGFI: adjusted goodness of fit index.

### 3.7 Direct, indirect and total influence of independent variables on dependent variables

**Fig. 2:**
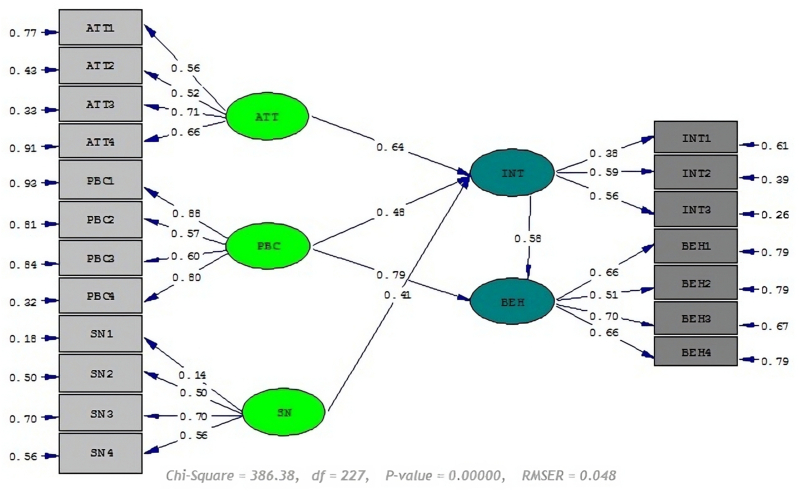
Structural equation model.

Using path analysis, the direct and indirect effects as well as the total effect of each of the independent variables on the dependent variables were shown. The results of this analysis are shown in Table 8 and Fig. 2. Moreover, the statistic of 5.44 shows that this relationship is significant at the level of 1.0 %.

**Table 8:** Structural model evaluation.

Dependent variable	independent variable	Direct effect	Indirect effect	Total effect	*t*-Value
Behavioral intention	Attitude	0.64	–	0.64	5.44^b^
	Subjective norm	0. 41	–	0.41	2.70^a^
	PBC	0.48	–	0.48	3.96^b^
Behavior	behavioral intention	0.58	–	0.58	10.03^b^
	PBC	0.79	0.28	1.07	6.80^b^

a Significant at 5.0 %.

b Significant at 1.0 %.

The direct effect of subjective norm on intention is estimated at 0.41 % and its t-statistic is 2.70, which indicates the significance of this relationship at the 5.0 % level. In addition, the direct effect of PBC on intention is estimated at 0.48 % and its *t-*statistic is 3.96, which indicates the significance of this relationship at the level of 1.0 %. The direct effect of behavioral intention on behavior is estimated at 0.58 % and its *t*-statistic is 10.03, which indicates the significance of this relationship at the level of 1.0 %. Finally, the direct effect of perceived behavior on behavior is 0.79 % and its *t*-statistic is estimated to be 6.80, which indicates the significance of this relationship at the 1.0 % level.

## 4 Discussion

The first step in the development of pesticide risk reduction programs is to determine the extent of the problem by examining the knowledge, attitude and behavior of farmers regarding agricultural pesticides [53]. According to the findings of this research, the average attitude showed the highest score (3.38), which indicates the positive to relatively neutral attitude of tomato growers towards the safe use of pesticides. However, the direct effect of tomato growers' attitude on their willingness was estimated with a coefficient of 0.58 and this relationship is significant at the 1 % level. The findings of this research were contrary to Bagheri et al.'s research in Iran. Their results show that 60 % of greenhouse owners had a relatively weak attitude towards the use of chemical pesticides [54], but it is consistent with the results of the study conducted by Damalas et al. [55]. False beliefs about pesticides and poisoning can significantly reduce the capacity of farmers and farm workers to protect themselves (occupational safety) against the dangers of pesticides [49]. Therefore, awareness of the attitude of farmers regarding the disposal of pesticide waste is a prerequisite for the development of intervention or prevention programs that should be targeted for safe ways of using pesticides, because the activity and decision of people towards any phenomenon is influenced by their attitude [56]. In general, people's more positive attitude towards a behavior can lead to more intention to perform that behavior [51]. Subjective norms showed a significant effect on the safe use of pesticides with an average overall score (2.34). The direct effect of mental norm on intention is estimated at 0.26 % and its *t*-statistic is 2.70, which indicates the significance of this relationship at the 5 % level. Studies conducted by Tama et al. similarly estimated the direct effect of mental norm on intention at 0.73 %, which indicates the significance of this relationship [57].

Subjective norms refer to a person's perceived social pressure (for example, friends, government employees or non-governmental organizations) and internal sources (for example, family and relatives) to perform or not perform a certain behavior [37]. This means that the greater the perceived social pressure, the greater the tendency to apply the subjective norm [48]. Thus, the positive and significant direct effects of mental norm on intention show that the perceived social pressure affects the intention of farmers [58]. Nouri et al. stated that the mental norm, attitude, and educational promotion have up to 45 % effect on the application of practical learning of villagers [59].

According to the findings of this research, there was a significant relationship between PBC and intention to use safely, and it showed the mean of 2.7. PBC is an important and effective factor in performing behavior [50]. If a person has strong control beliefs about the existence of facilitating factors of a behavior, he will have a high perceived control over the behavior [35]. On the contrary, if a person does not have strong control beliefs, he will have a low understanding of control, which prevents the occurrence of behavior [36,60]. In other words, the more farmers feel that they have more control over performing safe behaviors, the more they perform these behaviors. A low mean score of behavioral control relative to behavior indicates that farmers feel they have less control over safe behavior and it is in accordance with the findings of Bagheri et al. (2019) [61]. Regarding tomato growers' intention and actual behavior, as shown in Table 6, there was a significant relationship between tomato growers' intention and actual behavior for the safe use of pesticides (Pvalue = 0.005). So that tomato growers' intention on actual behavior caused the safe use of pesticides (as average = 2.57). Moreover, the intention for the safe use of pesticides was weak, while the respondents showed appropriate behavior with an average of (2.6) in the safe handling and spraying of pesticides. The results of the present study showed that behavioral intention is a predictor of tomato growers' safe behaviors in relation to safe use, which is in line with the studies of Rezaei (2019) [62] and Ataei (2021) and confirms the model of planned behavior [63]. The greater the intention of a person to perform unsafe behaviors, the riskier behaviors he shows. In fact, behavioral intention is an important predictor of behavior and should be taken into consideration in planning interventions to improve behavior towards the safe use of pesticides [64].

According to the findings of this research, most of the tomato growers, 86.7 %, had not participated in extension classes on how to dispose of the residual spraying solution, and most of them get this information experimentally and later through other farmers and pesticide sellers. Previous studies have shown that comprehensive hands-on training interventions significantly improve farmers' knowledge, especially for more complex pesticide characteristics that are difficult to learn empirically [65,66]. In this regard, adequate and reliable information sources can lead to a better understanding of risks and the use of preventive measures, even for people who do not have a high education [45,67]. Therefore, farmers dispose of pesticide residues based on their personal experience and do not know the correct and hygienic way, so poor behavior regarding the use, transfer and improper disposal of poisons is expected in this area. Creating awareness about the safe use of pesticides by a special program is very important [68]. In this study, 13 pesticides and 11 strengthening fertilizers were identified for tomato production, and none of them were very dangerous. On the other hand, compared with WHO toxicity classes, 100 % of pesticides contained moderately toxic, slightly toxic, and practically non-toxic compounds. Many more environmentally stable chemicals are widely used in developing countries, causing serious acute health problems and local and global environmental pollution [69].

The results of Andert's study showed that controlling weeds by delaying the planting date may be very effective to reduce the use of chemical pesticides by 50 % by 2030 [70]. In addition, the use of PPE when working with pesticides is also one of the important points that should be considered [52]. About 48 % of tomato growers in this study used PPE when loading and spraying pesticides, which is consistent with the results of the research conducted by Souza et al. [71]. Spraying clothing has the highest importance with 87 %, followed by glasses with 73.7 %. These findings are also consistent with Ajayi et al.'s study on the reasons why farmers do not use some PPE, the reasons of which are mostly related to the hot weather, high costs and lack of access to some protective equipment [72]. Regarding the disposal of the residual solution resulting from spraying, the results showed that 27.3 % of the pesticide residues are sprayed on the treated area and 51.1 % use it in another tomato field. These results are in line with the research of Damalas et al. (2008) - in their study they reported that farmers (30.2 %) usually use residual solutions on another product that is mentioned on the product label [28].

The tomato growers of the study stated that if the residual solution of some pesticides is repeatedly sprayed, their product may be weak or it may cause a layer of burn on the plant [73]. Therefore, the frequency of spraying should be reduced through extensive training in the field of Integrated Pest Management (IPM) [74]. In addition, most of the tomato growers (89 %) said that they dispose of the water from washing the sprayer tank and equipment in a part of the sprayed land where it is not close to surface water. Which was consistent with the study of Bagheri et al. (2021), they reported that (64.3 %) farmers leave the water from washing the sprayer tank in the field [18]. Braun et al. reported that three consecutive washings are enough for most formulations to remove 99 % of the pesticide remaining in containers [75]. Referring to the specified level, in Table 7, all indicators of the model's fitness are in a good condition. Table 7 shows that the GFI and AGFI values are 0.94 and 0.92, respectively. MacCallum & Hong suggested values above 0.9 for the GFI index has acceptable GFI [76]. Moreover, the values above 0.9 have been suggested for AGFI [77]. Therefore, our model has acceptable GFI and AGFI values.As reported in Table 7, the RMSEA value of our study is 0.048 that is the best value for RMSEA. Regarding RMSEA, values below 0.06 or even values below 0.07 and in the strictest case, the range between 0 and 0.05 are considered as the acceptance range of a good model fit [78,79]. Values above 0.1 indicate poor model fit [80].

## 5 Limitations

Among the research limits, dispersal of villages, time-consuming procedure for filling up the questionnaires and lack of financial support by the main institute, KUMS, where the Ethics Committee approved this research work, could be mentioned. Meanwhile, low cooperation of farmers and lack of their availability was one of the limits of the work. In addition, we considered those farmers having more than 1 ha cultivation land allocated to tomato. However, many farmers working on tomato growing at small pieces of farm, i.e. less than 1 ha were excluded from the study. There contributions might lead into different results or conclusion.

## 6 Conclusion

The results of this research show that the theory of planned behavior can be used as a suitable framework to encourage farmers to perform safe behaviors of pesticide waste disposal, and this means taking measures to strengthen the structures of the theory of planned behavior, especially behavioral intention and influential structures. On the intention, as it requires a perceived attitude and behavioral control in farmers, which can be done through actions such as formulating and implementing educational programs to raise awareness and create a positive attitude towards pesticide waste, involving farmers in educational programs to overcome perceived obstacles and problems. Improvement of proper waste disposal behavior is noted. Currently, according to the state of pesticide consumption, the production of these wastes is unavoidable, but the lack of proper management of the disposal of the residual spray solution after use and pesticide residues in the study area can cause great environmental and health risks. The necessity of using pesticides on the one hand and the emergence of health and environmental problems caused by their use on the other hand require that basic planning be done from the time of entry and production to consumption by considering different aspects of their impact. Therefore, the way of using pesticides, their environmental effects and their residual management in the environment are among the things that require special precision and planning.

## Suggestions

It is suggested that officials, planners, research institutes and agricultural faculties implement various educational programs by using new educational methods and holding different workshops. These programs include familiarization with actions after the use of poisons, the correct methods of pesticide waste disposal, and safety tips in the storage and maintenance of poison residues. The aim of these programs is to increase tomato growers' awareness of the dangers of improper disposal of pesticide waste and to teach them how to properly dispose of these wastes. In addition, direct communication between agricultural experts and promoters with farmers is recommended in order to change their attitude. Through holding training sessions and providing information in exchange environments, tomato growers can be taught how to use pesticide waste optimally and reduce their risks. In general, the preparation of brochures, radio and television programs, and the preparation of educational posters can also be effective in changing the attitude of tomato growers.

## CRediT authorship contribution statement

**Amin Pirmoghni:** Writing – original draft, Investigation, Data curation. **B. Shahmoradi:** Writing – review & editing, Supervision, Project administration, Conceptualization. **P. Taymoori:** Writing – review & editing, Software, Data curation. **Asghar Bagheri:** Visualization, Validation, Methodology, Investigation. **Parisa Nasrollahi:** Investigation, Data curation. **Zhino Karimi:** Investigation, Data curation. **Farough Mohammadian:** Writing – review & editing, Methodology. **Naier Emami:** Writing – review & editing, Validation, Conceptualization. **H.J. Choi:** Writing – review & editing, Supervision, Conceptualization.

## Declaration of competing interest

The authors declare that they have no known competing financial interests or personal relationships that could have appeared to influence the work reported in this paper.

## References

[1] Kosenchuk O., Shumakova O., Zinich A., Shelkovnikov S., Poltarykhin A. (2019). The development of agriculture in agricultural areas of Siberia: multifunctional character, environmental aspects. Journal of Environmental Management and Tourism.

[2] Balmford A., Green R.E., Scharlemann J.P. (2005). Sparing land for nature: exploring the potential impact of changes in agricultural yield on the area needed for crop production. Global Change Biol..

[3] Reimers K.J., Keast D.R. (2016). Tomato consumption in the United States and its relationship to the US Department of Agriculture food pattern: results from what We Eat in America 2005–2010. Nutr. Today.

[4] Fernandes P.B., Queiroz L.N., Michereff-Filho M., Cury N.F., Bonfim K., Cabral G.B. (2023). Expression of the Cry10Aa toxin in transgenic tomato confers tolerance to the tomato leafminer (Tuta absoluta). Sci. Hortic..

[5] Carvalho R., Duman K., Jones J.B., Paret M.L. (2019). Bactericidal activity of copper-zinc hybrid nanoparticles on copper-tolerant Xanthomonas perforans. Sci. Rep..

[6] Liu J., Li H., Yuan Z., Feng J., Chen S., Sun G. (2024). Effects of microbial fertilizer and irrigation amount on growth, physiology and water use efficiency of tomato in greenhouse. Sci. Hortic..

[7] Bjtsem Mw (1993).

[8] Cruz-López V., Granados-Echegoyen C.A., Pérez-Pacheco R., Robles C., Álvarez-Lopeztello J., Morales I. (2024). Plant diversity as a sustainable strategy for mitigating biotic and abiotic stresses in tomato cultivation. Front. Sustain. Food Syst..

[9] González-García Y., Cadenas-Pliego G., Alpuche-Solís Á.G., Cabrera R.I., Juárez-Maldonado A. (2021). Carbon nanotubes decrease the negative impact of Alternaria solani in tomato crop. Nanomaterials.

[10] Gatahi D.M. (2020). Challenges and opportunities in tomato production chain and sustainable standards. Int. J. Hortic. Sci. Technol..

[11] Ansari M., Taghavi S.M., Hamzehzarghani H., Valenzuela M., Siri M.I., Osdaghi E. (2019). Multiple introductions of tomato pathogen Clavibacter michiganensis subsp. michiganensis into Iran as revealed by a global-scale phylogeographic analysis. Appl. Environ. Microbiol..

[12] Hejazi M., Movahedi M., Askari O., Higbee B. (2016). Novel chemo-attractants for trapping tomato leafminer moth (Lepidoptera: gelechiidae). Journal of economic entomology.

[13] Peritore-Galve F.C., Tancos M.A., Smart C.D. (2021). Bacterial canker of tomato: revisiting a global and economically damaging seedborne pathogen. Plant Dis..

[14] Pijnakker J., Moerkens R., Vangansbeke D., Duarte M., Bellinkx S., Benavente A. (2022). Dual protection: a tydeoid mite effectively controls both a problem pest and a key pathogen in tomato. Pest Manag. Sci..

[15] Hu F.-Y., Mou D.-F., Tsai C.-W. (2020). Evaluation of barrier plants for the cultural control of tomato yellow leaf curl disease. J. Asia Pac. Entomol..

[16] Leili M., Pirmoghani A., Samadi M.T., Shokoohi R., Roshanaei G., Poormohammadi A. (2016). Determination of pesticides residues in cucumbers grown in greenhouse and the effect of some procedures on their residues. Iranian journal of public health.

[17] Arias L.A., Garzón A., Ayarza A., Aux S., Bojacá C.R. (2021). Environmental fate of pesticides in open field and greenhouse tomato production regions from Colombia. Environmental Advances.

[18] Bagheri A., Pirmoazen S., Allahyari M.S. (2021). Assessment of farmers' understanding of the pictograms displayed on pesticide labels. Environ. Sci. Pollut. Control Ser..

[19] Rajak P., Roy S., Ganguly A., Mandi M., Dutta A., Das K. (2023). Agricultural pesticides–friends or foes to biosphere?. Journal of Hazardous Materials Advances.

[20] Saroop S., Tamchos S. (2024). Impact of pesticide application: positive and negative side. Pesticides in a Changing Environment.

[21] Felix M., Holst N., Sharp A. (2019). PestTox: an object oriented model for modeling fate and transport of pesticides in the environment and their effects on population dynamics of non-target organisms. Comput. Electron. Agric..

[22] Sachan S.G. (2023). Current Developments in Biotechnology and Bioengineering.

[23] Karunarathne A., Gunnell D., Konradsen F., Eddleston M. (2020). How many premature deaths from pesticide suicide have occurred since the agricultural Green Revolution?. Clinical toxicology.

[24] Staudacher P., Fuhrimann S., Farnham A., Mora A.M., Atuhaire A., Niwagaba C. (2020). Comparative analysis of pesticide use determinants among smallholder farmers from Costa Rica and Uganda. Environ. Health Insights.

[25] Cui N., Ba X., Dong J., Fan X. (2022). Does farmland transfer contribute to reduction of chemical fertilizer use? evidence from Heilongjiang Province, China. Sustainability.

[26] Kapeleka J.A., Sauli E., Sadik O., Ndakidemi P.A. (2019). Biomonitoring of acetylcholinesterase (AChE) activity among smallholder horticultural farmers occupationally exposed to mixtures of pesticides in Tanzania. Journal of environmental and public health.

[27] Damalas C.A., Eleftherohorinos I.G. (2011). Pesticide exposure, safety issues, and risk assessment indicators. Int. J. Environ. Res. Publ. Health.

[28] Damalas C.A., Telidis G.K., Thanos S.D. (2008). Assessing farmers' practices on disposal of pesticide waste after use. Science of the total environment.

[29] Sugavanam B. (1996). Risk reduction in pesticide development in developing countries‐challenges and opportunities. Journal of Environmental Science & Health Part B.

[30] Faryabi R., Mokhtari M., Rahimi T., Javadi A., Rastegari N. (2017). Investigation of status and correlations between Knowledge, Attitude and Performance of Greenhouse Farmers of Jiroft Township in relation to adverse health and environmental effects of the use of pesticides in 2015. Iran. Occup. Health.

[31] Sandoghi A., Yousefi A., Amini A. (2015). Evaluation of factors affecting cucumber-and-tomato greenhouse farmers' attitudes toward healthy crops production in Isfahan Township. Journal of Science and Technology of Greenhouse Culture.

[32] Sandy Y.A., Zahro F.A., Rizky D.R., Fajarwati S.K., Effendi M. (2024). Knowledge level of farmers regarding the use of pesticide for pest and disease control. Jurnal Agrinika: Jurnal Agroteknologi dan Agribisnis.

[33] Abaineh A., Ejigu D., Atlabachew M., Dejen E., Tilahun G. (2023). Knowledge, attitude and practices of farmers and experts about the effects of pesticide residues on agricultural product users and ecosystems: a case of Fogera District, Ethiopia. PLoS One.

[34] Berni I., Menouni A., El I.G., Duca R.-C., Kestemont M.-P., Godderis L. (2021). Understanding farmers' safety behavior regarding pesticide use in Morocco. Sustain. Prod. Consum..

[35] Colémont A., Van den Broucke S. (2008). Measuring determinants of occupational health related behavior in Flemish farmers: an application of the theory of planned behavior. J. Saf. Res..

[36] Ajzen I. (1991). The theory of planned behavior. Organ. Behav. Hum. Decis. Process..

[37] Bagheri A., Bondori A., Allahyari M.S., Damalas C.A. (2019). Modeling farmers' intention to use pesticides: an expanded version of the theory of planned behavior. J. Environ. Manag..

[38] Menozzi D., Sogari G., Mora C. (2015). Explaining vegetable consumption among young adults: an application of the theory of planned behaviour. Nutrients.

[39] Bond J., Kriesemer S., Emborg J., Chadha M. (2009). Understanding farmers' pesticide use in Jharkhand India. Extension Farming Systems Journal.

[40] Wang J., Chu M., Deng Yy, Lam H., Tang J. (2018). Determinants of pesticide application: an empirical analysis with theory of planned behaviour. China Agric. Econ. Rev..

[41] Fielding K.S., Terry D.J., Masser B.M., Hogg M.A. (2008). Integrating social identity theory and the theory of planned behaviour to explain decisions to engage in sustainable agricultural practices. Br. J. Soc. Psychol..

[42] Monfared N., Yazdanpanah M., Tavakoli K. (2018).

[43] Jamshidi A., Morovati M., Golbini Mofrad M.M., Panahandeh M., Soleimani H., Abdolahpour Alamdari H. (2021). Water quality evaluation and non-cariogenic risk assessment of exposure to nitrate in groundwater resources of Kamyaran, Iran: spatial distribution, Monte-Carlo simulation, and sensitivity analysis. Journal of Environmental Health Science and Engineering.

[44] Farashi Z., Mirdrikvand M., Shanazi K., Gholamrezai S. (2021). Analysing the health behavior in use of chemical pesticides (case: farmers of khorramabad county). Iran. J. Agric. Econ. Dev. Res..

[45] Sharafi K., Pirsaheb M., Maleki S., Arfaeinia H., Karimyan K., Moradi M. (2018). Knowledge, attitude and practices of farmers about pesticide use, risks, and wastes; a cross-sectional study (Kermanshah, Iran). Science of the total environment.

[46] Bagheri A., Emami N., Damalas C.A. (2021). Farmers' behavior towards safe pesticide handling: an analysis with the theory of planned behavior. Science of the total Environment.

[47] Bondori A., Bagheri A., Allahyari M.S., Damalas C.A. (2019). Pesticide waste disposal among farmers of Moghan region of Iran: current trends and determinants of behavior. Environ. Monit. Assess..

[48] Wang J., Chu M., yuan Deng Y., Lam H., Tang J. (2018). Determinants of pesticide application: an empirical analysis with theory of planned behaviour. China Agric. Econ. Rev..

[49] Ibitayo O.O. (2006). Egyptian farmers' attitudes and behaviors regarding agricultural pesticides: implications for pesticide risk communication. Risk Anal..

[50] Li J., Jiang R., Tang X. (2024). Assessing psychological factors on farmers' intention to apply organic manure: an application of extended theory of planned behavior. Environ. Dev. Sustain..

[51] Gao L., Wang S., Li J., Li H. (2017). Application of the extended theory of planned behavior to understand individual's energy saving behavior in workplaces. Resour. Conserv. Recycl..

[52] Indriati G. (2022). The relationship of knowledge, attitudes, and actions with the completeness of the use of personal protective equipment (PPE) in farmers insecticide. Jurnal Penelitian Pendidikan IPA.

[53] Damalas C.A., Koutroubas S.D., Abdollahzadeh G. (2024). Farmers' willingness to use lower risk pesticides for pest control: barriers and facilitators in northern Greece. Environmental Challenges.

[54] Bagheri A., Shirzadi Z., Bondori A., Sawari Mobini A. (2024). Factors affecting hygiene behavior of greenhouse owners of khozestan on pesticides. Journal of Environmental Science Studies.

[55] Damalas C.A. (2021). Farmers' intention to reduce pesticide use: the role of perceived risk of loss in the model of the planned behavior theory. Environ. Sci. Pollut. Control Ser..

[56] Owemigisha E., Rwot A.O., Sempebwa D., Birungi C.M., Tamale A., Drileyo G. (2024). Exploring knowledge, attitudes and practices of farmers at the edge of Budongo forest on agrochemicals usage. Sustainable Environment.

[57] Tama R.A.Z., Ying L., Yu M., Hoque M.M., Adnan K.M., Sarker S.A. (2021). Assessing farmers' intention towards conservation agriculture by using the Extended Theory of Planned Behavior. J. Environ. Manag..

[58] Hou B., Wang Z-w, Ying R. (2016). Pesticide residues and wheat farmer's cognition: a China scenario. Agricultural research.

[59] Noori S.H.A., Alavion S.J. (2016).

[60] Waichman A.V., Eve E., da Silva Nina N.C. (2007). Do farmers understand the information displayed on pesticide product labels? A key question to reduce pesticides exposure and risk of poisoning in the Brazilian Amazon. Crop Protect..

[61] Bagheri A., Emami N., Damalas C.A., Allahyari M.S. (2019). Farmers' knowledge, attitudes, and perceptions of pesticide use in apple farms of northern Iran: impact on safety behavior. Environ. Sci. Pollut. Control Ser..

[62] Rezaei R., Seidi M., Karbasioun M. (2019). Pesticide exposure reduction: extending the theory of planned behavior to understand Iranian farmers' intention to apply personal protective equipment. Saf. Sci..

[63] Ataei P., Gholamrezai S., Movahedi R., Aliabadi V. (2021). An analysis of farmers' intention to use green pesticides: the application of the extended theory of planned behavior and health belief model. J. Rural Stud..

[64] Abdollahzadeh G., Damalas C.A., Sharifzadeh M.S., Ahmadi-Gorgi H. (2018).

[65] Goeb J., Lupi F. (2021). Showing pesticides' true colors: the effects of a farmer-to-farmer training program on pesticide knowledge. J. Environ. Manag..

[66] Boateng K.O., Dankyi E., Amponsah I.K., Awudzi G.K., Amponsah E., Darko G. (2023). Knowledge, perception, and pesticide application practices among smallholder cocoa farmers in four Ghanaian cocoa-growing regions. Toxicol Rep.

[67] Salameh P.R., Baldi I., Brochard P., Abi Saleh B. (2004). Pesticides in Lebanon: a knowledge, attitude, and practice study. Environ. Res..

[68] Karunamoorthi K., Mohammed M., Wassie F. (2012). Knowledge and practices of farmers with reference to pesticide management: implications on human health. Arch. Environ. Occup. Health.

[69] Ecobichon D.J. (2001). Pesticide use in developing countries. Toxicology.

[70] Andert S., Ziesemer A., de Mol F. (2024). The link between farmers' sowing date and herbicide management. Crop Protect..

[71] Lessa de Souza A., Viviana Waichman A. (2024). BEHAVIOUR, practices and attitudes of farmers regarding the use of pesticides at the agricultural frontier in the south of the amazon state. Environmental & Social Management Journal/Revista de Gestão Social e Ambiental.

[72] Ajayi O.C., Akinnifesi F.K. (2007). Farmers' understanding of pesticide safety labels and field spraying practices: a case study of cotton farmers in northern Côte d'Ivoire. Sci. Res. Essays.

[73] Parween T., Jan S., Mahmooduzzafar S., Fatma T., Siddiqui Z.H. (2016). Selective effect of pesticides on plant—a review. Crit. Rev. Food Sci. Nutr..

[74] Kishi M., Hirschhorn N., Djajadisastra M., Satterlee L.N., Strowman S., Dilts R. (1995). Relationship of pesticide spraying to signs and symptoms in Indonesian farmers. Scand. J. Work. Environ. Health.

[75] Braun H., Morrow D., Ripley B., Frank R. (1983). Efficiency of water rinsing for the decontamination of used pesticide containers. Arch. Environ. Contam. Toxicol..

[76] MacCallum R.C., Hong SJMbr (1997). Power analysis in covariance structure modeling using GFI and AGFI.

[77] Hooper D.C.J., Mullen M.R. (2008). Structural equation modelling: guidelines for determining model fit. Electron. J. Bus. Res. Methods.

[78] Lt Hu, Pmjsemamj Bentler (1999). Cutoff criteria for fit indexes in covariance structure analysis: conventional criteria versus new alternatives.

[79] Steiger J.H.J.P., differences I. (2007). Understanding the limitations of global fit assessment in structural equation modeling.

[80] Hoyle R.H., Davisson E.K., Diebels K.J., Leary M.R.J.P., Differences I. (2016).

